# Assessment of a nanocrystal 3-D morphology by the analysis of single HAADF-HRSTEM images

**DOI:** 10.1186/1556-276X-8-475

**Published:** 2013-11-13

**Authors:** Daniel G Stroppa, Ricardo D Righetto, Luciano A Montoro, Lothar Houben, Juri Barthel, Marco AL Cordeiro, Edson R Leite, Weihao Weng, Christopher J Kiely, Antonio J Ramirez

**Affiliations:** 1 Brazilian Nanotechnology National Laboratory, Campinas, São Paulo 13083-970, Brazil; 2 Ernst Ruska Centre, Forschungszentrum Jülich GmbH, Jülich 52425, Germany; 3 Mechanical Engineering School, University of Campinas, Campinas, São Paulo 13083-860, Brazil; 4 School of Electrical and Computer Engineering, University of Campinas, Campinas, São Paulo 13083-852, Brazil; 5 Department of Chemistry, Federal University of São Carlos, São Carlos 13565-905, Brazil; 6 Department of Materials Science and Engineering, Lehigh University, Bethlehem, PA 18015, USA

**Keywords:** 3-D morphology, HAADF, HRSTEM, Nanocrystal modeling

## Abstract

This work presents the morphological characterization of CeO_2_ nanocrystals by the analysis of single unfiltered high-angle annular dark-field (HAADF)-high-resolution scanning transmission electron microscopy (HRSTEM) images. The thickness of each individual atomic column is estimated by the classification of its HAADF integrated intensity using a Gaussian mixture model. The resulting thickness maps obtained from two example nanocrystals with distinct morphology were analyzed with aid of the symmetry from the CeO_2_ crystallographic structure, providing an approximation for their 3-D morphology with high spatial resolution. A confidence level of ±1 atom per atomic column along the viewing direction on the thickness estimation is indicated by the use of multislice image simulation. The described characterization procedure stands out as a simple approach for retrieving morphological parameters of individual nanocrystals, such as volume and specific surface areas for different crystalline planes. The procedure is an alternative to the tilt-series tomography technique for a number of nanocrystalline systems, since its application does not require the acquisition of multiple images from the same nanocrystal along different zone axes.

## Background

Elucidating the morphology of nanostructured materials with high resolution is essential for their optimization for specific applications. A remarkable example is the use of nanostructured materials in catalysis, as their performance often depends on the exposed facet crystallographic structure and surface areas [1,2]. Even though significant efforts have been devoted to the three-dimensional (3-D) morphology characterization of individual nanocrystals [3,4], a straightforward and undemanding method is still unavailable.

The high-angle annular dark-field (HAADF) imaging mode in high-resolution scanning transmission electron microscopy (HRSTEM) [5-7] is one of the most promising techniques for nanocrystal characterization. Besides allowing direct imaging of the atomic columns with a spatial resolution down to 50 pm [7], the high-angle scattered electron signal intensity can be directly correlated to the thickness of the atomic columns [8] and the atomic number of their constituent atoms [9-11].

Recent reports have shown the successful application of HAADF-HRSTEM for mapping the thickness of Au foils [12] and for reconstructing the 3-D morphology with atomic resolution of an Ag precipitate in an Al matrix after applying a tomographic reconstruction procedure [13]. These studies benefit from the use of HRSTEM multislice image simulations [14,15] and Gaussian mixture models (GMMs) [16] to relate the integrated signal of each atomic column to their thickness with high accuracy. However, the requirement of model samples and/or the acquisition of multiple images from the same nanocrystal along different zone axes may restrict the practical application of these methodologies, especially for those systems that cannot withstand a high electron dose. In addition, sample and microscope instabilities during sample tilting procedures may make the 3-D morphology characterization of nanocrystalline samples by tomography approaches very difficult or even impractical. Consequently, the determination of the 3-D structure from nanoparticles by the use of a single electron microscopy image is a current challenge, which has been accomplished so far only for very specific systems such as size-selected gold clusters [17] and thin layers of light-weight atoms [18].

This work presents an alternative approach for estimating the 3-D morphology of nanocrystalline samples by the analysis of single HAADF-HRSTEM images. The described methodology combines two steps, namely (1) the determination of the thickness of each atomic column by GMM classification and (2) the construction of a 3-D morphology model using crystallographic symmetry operations which define the atom positions in the unit cell of the examined sample.

We applied the method to faceted CeO_2_ nanocrystals displaying two different morphologies [19]. The CeO_2_ nanocrystalline system was chosen for this study because of two characteristics. First, CeO_2_-based materials show outstanding catalytic properties depending on the exposed facets, their surface area, and crystallographic structure [20]. Second, the highly regular faceting present on the studied CeO_2_ nanocrystals allows a quantitative comparison between experimental results and HRSTEM image simulations based on symmetric model structures, which in turn provides us with an evaluation of the accuracy of thickness determination. However, the method requires neither the presence of faceted nanocrystals nor the use of image simulation procedures.

## Methods

CeO_2_ nanocrystals were synthesized following a previously reported two-phase approach [21]. In this method, an aqueous solution of cerium(III) nitrate (30 mL, 0.085 mol/L) was transferred to a 100-mL Teflon-lined stainless steel autoclave, and then toluene (30 mL) and *tert*-butylamine (0.30 mL) were added under ambient conditions without stirring. CeO_2_ nanocrystals with distinctly differing morphologies could be generated by manipulating the oleic acid (OA) concentration employed in the preparation. Polyhedral (labeled type-A) and cube-like (labeled type-B) CeO_2_ nanocrystals were obtained by the use of low (3 mL) and high (6 mL) OA additions, respectively. A thermal treatment at 180°C for 24 h was carried out for both variants of the synthesis process. Finally the material was washed several times and re-dispersed in non-polar solvents (e.g., toluene, hexane, chloroform) after the reaction.

Samples for electron microscopy analysis were prepared by dropping the diluted colloidal solution onto copper grids covered with a thin (approximately 5 nm) continuous amorphous carbon film and allowing the solvent to evaporate. STEM characterization was carried out using a JEOL JEM-2200FS microscope (JEOL Ltd., Akishima, Tokyo, Japan) equipped with a probe corrector and a Schottky field-emission electron gun operating at 200 kV. The HRSTEM imaging experiments were carried out using an electron beam with a 25-mrad convergence angle, 0.09-nm spot size, and a 64 μs/pixel dwell time during scanning. The HAADF signal within the 110- to 330-mrad angular range was acquired simultaneously to the bright-field (BF) signal.

The detection of peaks associated with atomic columns and their signal integration were performed for the 'as-obtained’ HAADF-HRSTEM images using circular masks with fixed radius. The size of the masks was selected to include approximately 80% of the peak intensities. A detailed description of these procedures can be found in Additional file 1.

The atomic column thickness estimation procedure was carried out separately for type-A and type-B nanocrystal images. The integrated intensities obtained for the individual atomic columns were classified as a histogram, and the data fitting procedure was performed by the use of multiple normal distributions according to the GMM [16]. Multiple runs of the GMM algorithm were performed in order to optimize the model parameters, which were the number of Gaussian fitting distributions and their respective mean values, amplitudes, and standard deviations. The optimum GMM configuration was selected so that the absolute value of residual error between the model and the dataset was minimized.

After the GMM optimum parameter determination, each atomic column thickness was assigned to a Gaussian distribution. The correlation between the fitting distributions and the atomic column thicknesses was performed by taking into account (1) the monotonic increase of the mean value of the fitting curves and (2) the integrated intensity of the isolated Ce atoms. The integrated intensity analysis from isolated Ce atoms can be found in Additional file 1.

Finally, the spacing between Ce atoms along the imaging zone axes was evaluated for both type-A and type-B nanocrystals according to the allowed symmetry operations of the CeO_2_ unit cell. This approach led to the 3-D morphology models of the examined nanocrystals and to their geometrical parameter quantification. Atomic structure files of the 3-D morphology models are available in Additional files 2 and 3.

## Results and discussion

**Figure 1:**
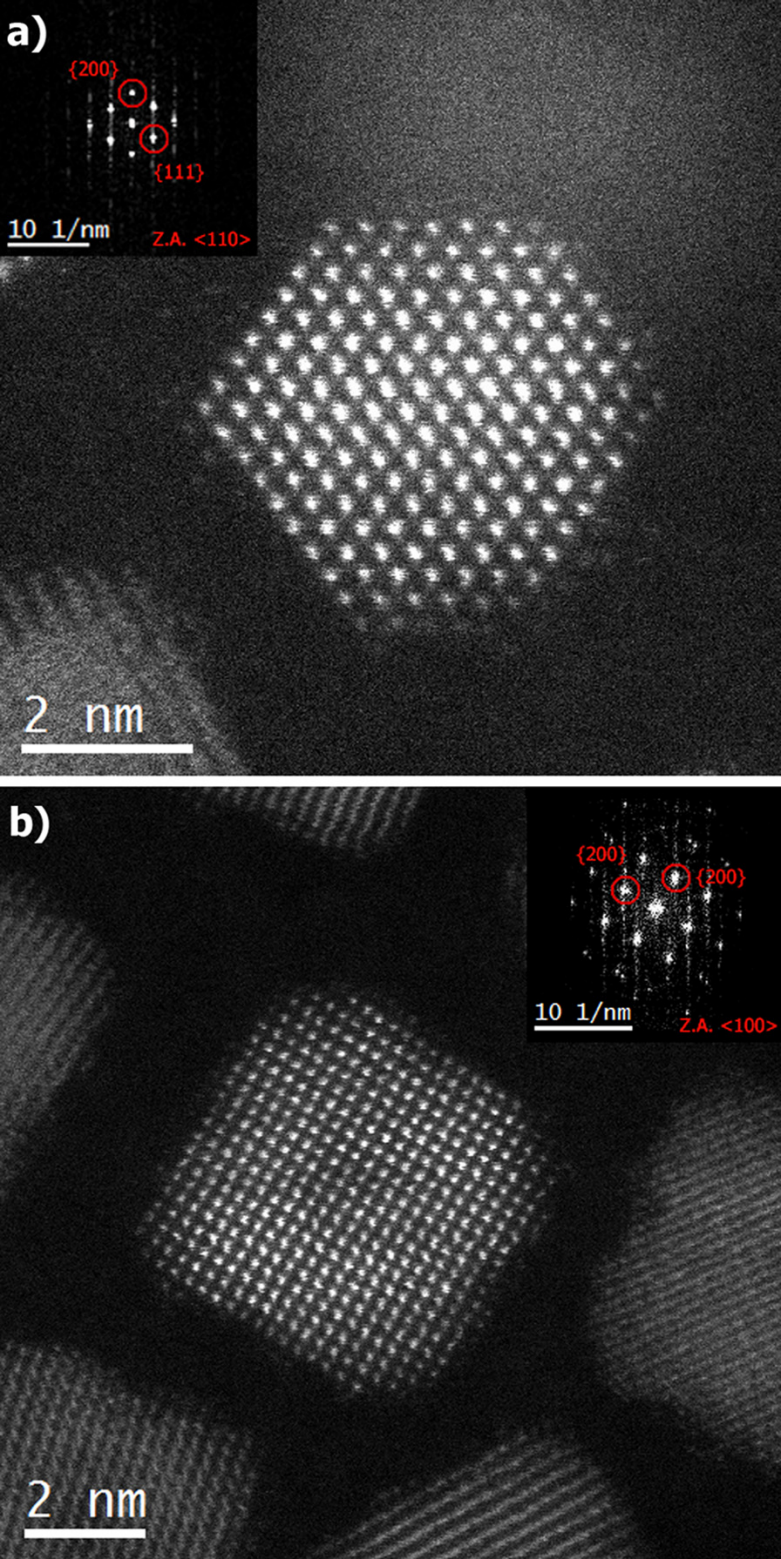
**HAADF-HRSTEM images from (a) type-A and (b) type-B CeO**_**2 **_**nanocrystals.** As-obtained HAADF-HRSTEM images from **(a)** type-A and **(b)** type-B CeO_2_ nanocrystals. The insets show the images' FT analysis, including the zone axes and the projected crystallographic plane indexing.

Figure 1 presents representative HAADF-HRSTEM images of CeO_2_ nanocrystals, which were synthesized with a low (Figure 1a) and a high (Figure 1b) oleic acid concentration. These particles are representative for the polyhedral type-A and cube-like type-B nanocrystals present in the studied samples. Due to the atomic weight dependence in the high-angle electron scattering regime and the experimental imaging configuration, only the Ce atomic columns present a distinguishable signal with respect to the background. The insets in Figure 1 correspond to the Fourier transform (FT) of the images, which were used to determine the nanocrystal zone axis orientation and the projected atomic plane indexes. Although the crystalline plane indexation indicates {111} and {200} terminations for the type-A nanocrystal and {220} and {200} terminations for the type-B nanocrystal, it is not possible to unambiguously evaluate the particles' 3-D morphology from the crystallographic analysis alone due to the projection characteristic inherent to the TEM imaging.

**Figure 2:**
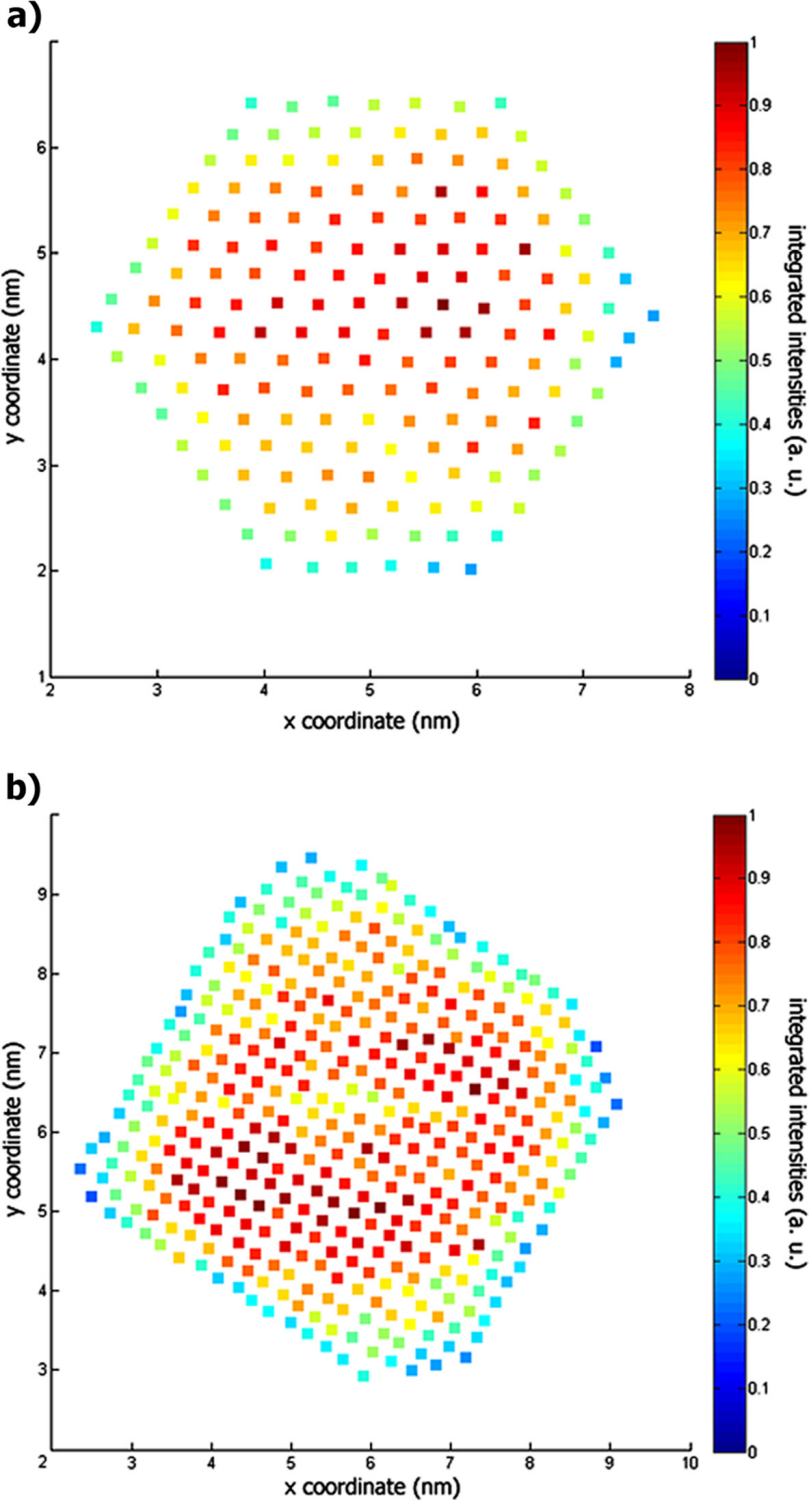
**Color maps with signal integration from (a) type-A and (b) type-B nanocrystals' individual atomic columns.** Color maps obtained after the signal integration of individual atomic columns from the images of type-A and type-B nanocrystals, respectively. The color coding refers to the integrated intensity from the unfiltered HAADF-HRSTEM images presented in Figure 1. The values were normalized to the atomic column with the highest integrated intensity in each image.

In order to establish the three-dimensional shape of the particles, the quantitative analysis of these two particular HAADF-HRSTEM images started with the atomic column peak detection and signal integration. Figure 2 shows color maps depicting the intensity of the integrated signal for each atomic column within type-A and type-B nanocrystal images.

Although the morphology of the nanocrystals can be roughly inferred from the integrated signal intensity of the columns and from the indexing of the projected crystallographic planes, a more accurate analysis can be achieved from a column-by-column thickness evaluation using the assumption that the number of Ce atoms in each column is an integer. Then, any unwanted contributions to the atomic column signal from other sources, such as the inherent (1) noise of the detection system, (2) electron channeling effects, and the (3) background HAADF signal from the oxygen atoms in the CeO_2_, the carbon support film, and the ligand molecules attached to the nanoparticles, are approximated to nearest value for the HAADF scattering of a discrete Ce atomic column.

**Figure 3:**
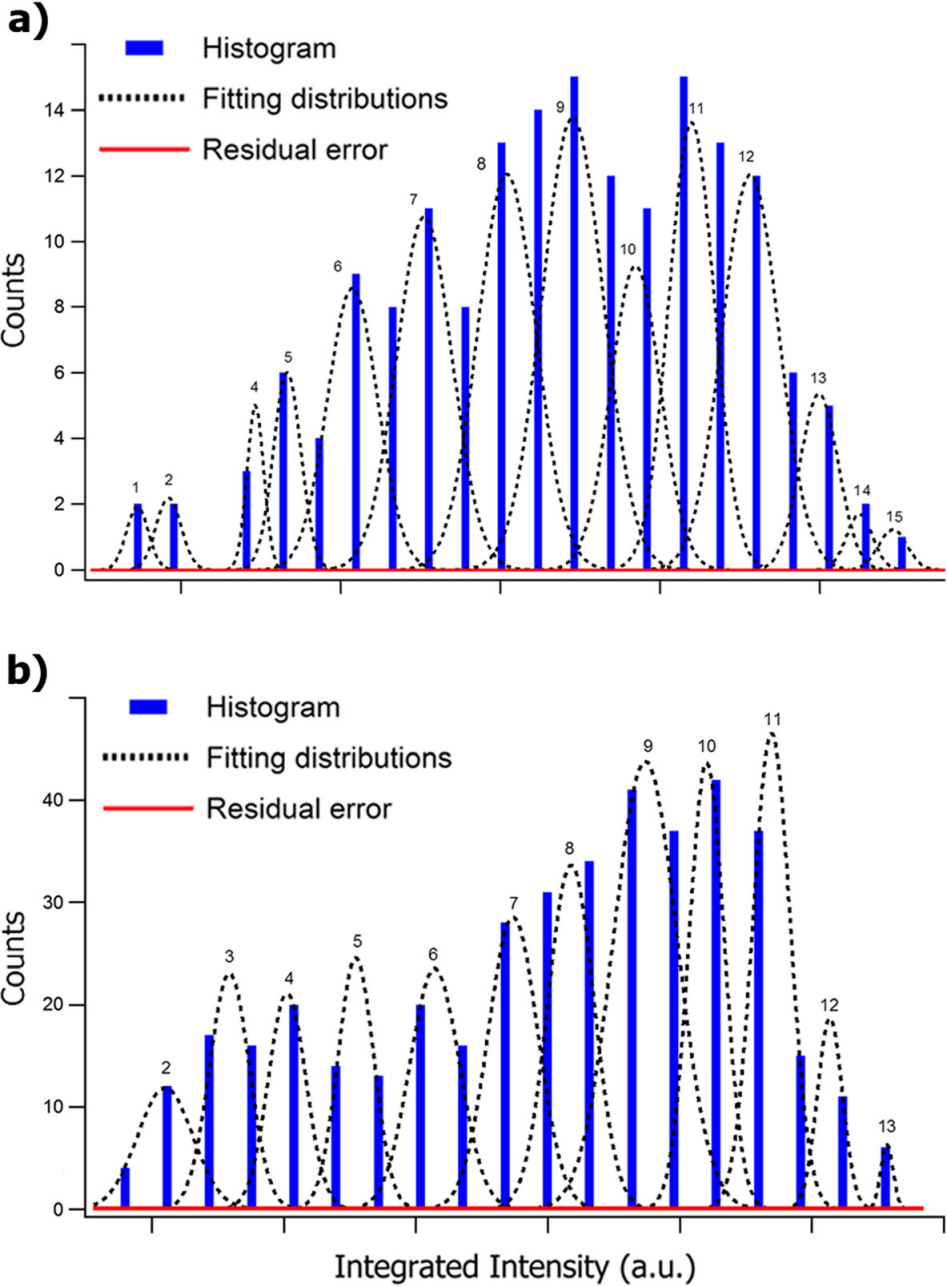
**Integrated intensity histograms and GMM classification from (a) type-A and (b) type-B nanocrystals.** Integrated peak intensity histograms derived from the HAADF-HRSTEM images of **(a)** type-A and **(b)** type-B CeO_2_ nanocrystals. The dashed lines indicate the optimal distribution curves after the GMM fitting, and the solid red curves indicate the residual errors. The number assigned to each distribution curve refers to the number of Ce atoms along the projected atomic columns.

Figure 3 shows histograms of the integrated peak intensities from Figure 2, including the optimized GMM configurations obtained following the procedure described on the previous section, the residual errors for the analyzed datasets, and the quantized column thickness assigned to each Gaussian distribution.

The correlation between the distribution curves and the number of Ce atoms on the analyzed columns presented in Figure 3 was obtained from the analysis of two relevant aspects. The first is the monotonic increase of the mean values of the Gaussian distributions, as expected for an increasing number of atoms on the projected columns [13]. The second parameter is the integrated intensity of the peripheral atomic positions in each HAADF image, which supports the intensity distribution assignment for a single Ce atom.

The standard deviation parameter from each GMM distribution curve is related to the overall noise contributions to the HAADF signal. However, the extent of such effects cannot be quantitatively analyzed from the fitting results due to the low occurrence of some atomic column lengths in the evaluated samples. Nevertheless, the thickness assignment precision can be conservatively estimated to be ±1 atom from the maximum overlap of the distribution curves, given the optimization of the Gaussian distribution standard deviations after the GMM fitting.

**Figure 4:**
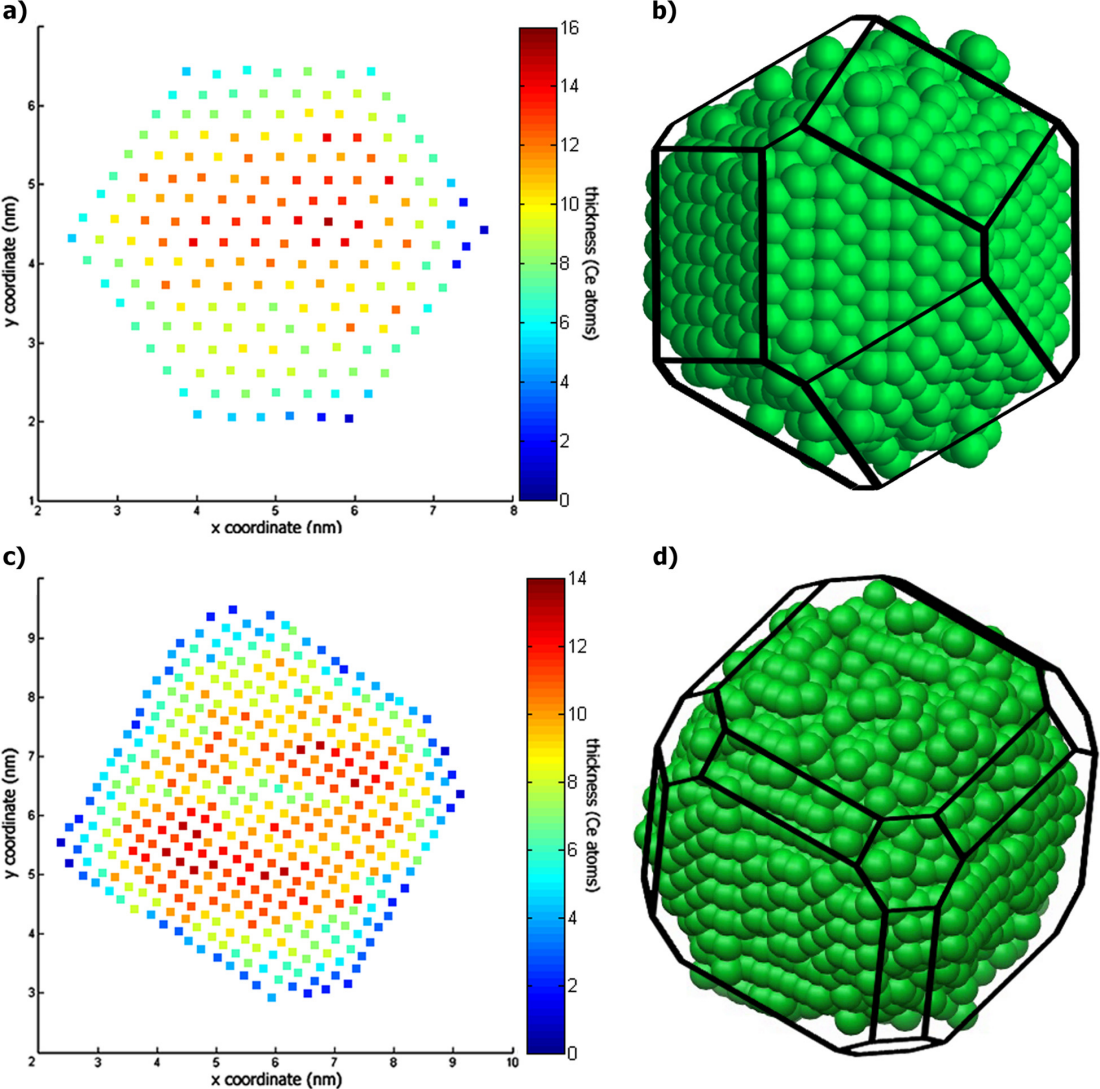
**Color maps and 3-D morphology models based on thickness maps obtained by the GMM fitting. (a, c)** Color maps generated after thickness map quantization by the GMM fitting procedure for type-A and type-B nanocrystals, respectively. The color coding now represents the number of Ce atoms along each atomic column position. **(b, d)** 3-D morphology models based on the calculated column thicknesses and on the symmetry operations allowed by the CeO_2_ crystalline structure.

The quantized thickness maps for both CeO_2_ nanocrystals with A- and B-type morphologies are shown in Figure 4a,c, respectively. With the aid of crystallographic symmetry operations, the corresponding 3-D morphology models from nanocrystals A and B were generated, as depicted in Figure 4b,d, respectively. The atomic coordinates in the 3-D morphology models were calculated considering the zone axis orientation observed in the experimental HAADF-HRSTEM images, and from the mirror operations that are allowed for both the 〈100〉 and 〈110〉 direction families according to the Fm3m symmetry of the CeO_2_ unit cell. It should be noted that the green spheres in Figure 4b,d which represent Ce atoms in the nanocrystals are illustrative in that their radii and positions along the zone axis are not in complete agreement with the unit cell structure. A movie and an interactive 3-D morphology model for each analyzed CeO_2_ nanocrystal are available in Additional files 4, 5, 6, 7.

The 3-D morphology models successfully resolve structural features in accordance with projected facets on the HAADF-HRSTEM images, such as the {111} and {200} terminations for type-A nanocrystal in Figure 1a and {111}, {220}, and {200} terminations for type-B nanocrystal in Figure 1b. In addition, their projections along different zone axes provide the average nanocrystal dimensions for different crystallographic directions, as indicated by the superimposed wireframe structure in Figure 4b,d. Consequently, a reasonable approximation for the total volume and the surface areas from different crystallographic facets can be retrieved from the 3-D morphology models. The geometric parameters calculated from this analysis are summarized in Table 1.

**Table 1:** Calculated total surface areas of specific facets from the 3-D morphology models

	**Facet**	**Surface area (nm**^**2**^**)**	**Volume (nm**^**3**^**)**
Nanocrystal A	{200}	30.1	54.3
	{111}	45.2	
Nanocrystal B	{200}	105.1	146.0
	{220}	34.9	
	{111}	9.4	

The symmetry operations of the unit cell effectively apply to the morphology and also to the growth behavior general descriptions from several nanostructured systems [22]. In such cases, the quantification of HAADF-HRSTEM images for the evaluation of the thickness of atomic columns and the use of symmetry operations to estimate the 3-D morphology is well founded. It may, however, be unrealistic for cases with element segregation that generates considerable *Z* contrast, such as core-shell structures and porous and/or non-convex structures, for example.

In order to further verify the adequacy of the models describing the CeO_2_ nanocrystalline systems evaluated here, HAADF-HRSTEM image simulations were carried out for symmetric model structures based on the 3-D morphology models presented in Figure 4, and the results were compared to the experimental images. The precise structures used in the image simulation [23], the simulation parameters, the corresponding simulated HAADF-HRSTEM images, and the qualitative comparisons between the simulated and experimental images are available in Additional files 1, 8 and 9.

**Figure 5:**
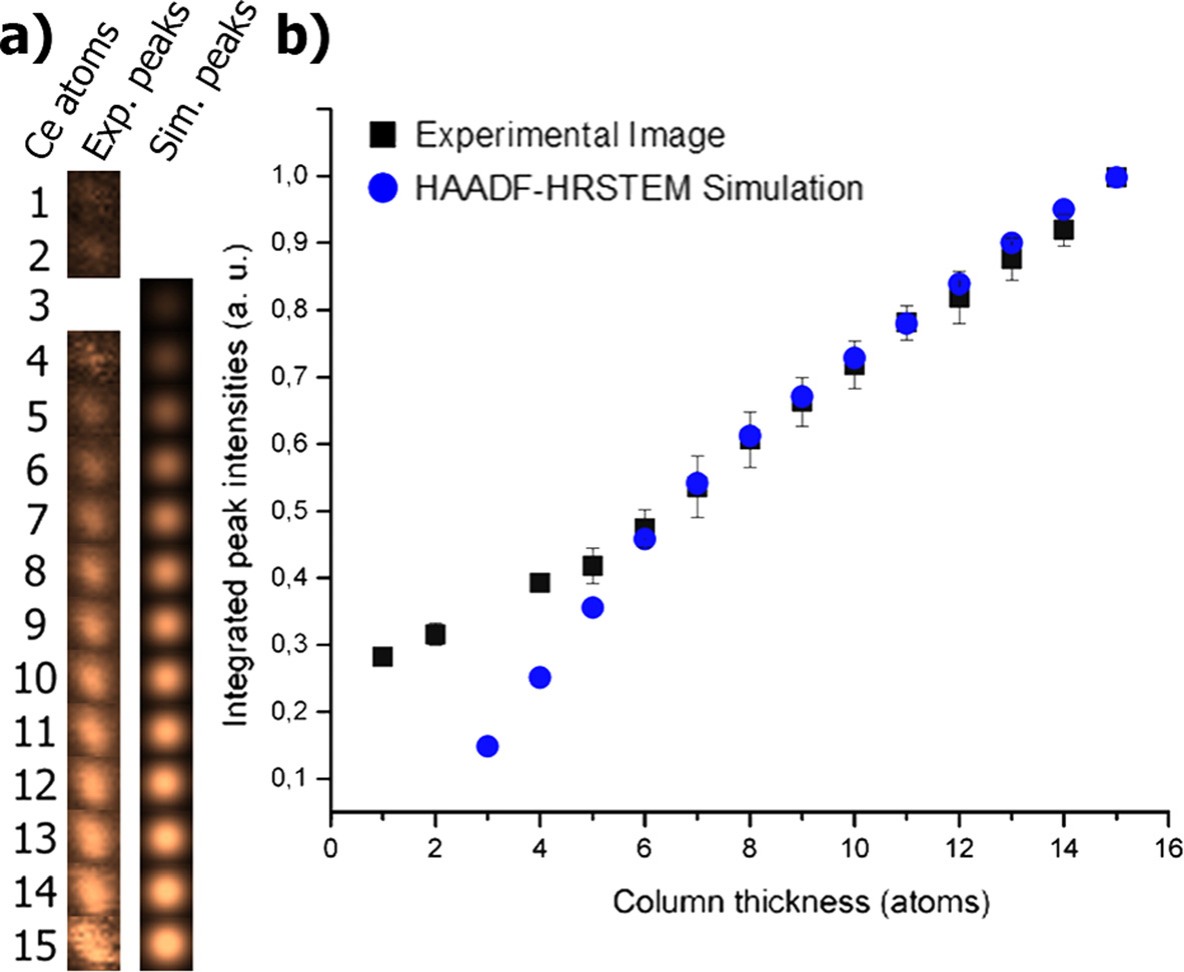
**Comparison between experimental and simulated average peak images for different atomic column thicknesses. (a)** Comparison between experimental and simulated average peak images for different atomic column thicknesses. The experimental data were taken from the image of the type-A nanocrystal. **(b)** Comparison between the normalized intensity values for experimental and simulated image peaks.

The quantitative comparison between the experimental and simulated HAADF-HRSTEM images demonstrates a direct correlation of the integrated intensities of the atomic column signal in the experiment and simulation, as shown in Figure 5 for nanocrystal A. Figure 5a presents a fair agreement between the experimental and simulated average peak images for different column thicknesses. The minor differences between the two datasets can be justified in terms of a number of factors which were not taken into account in the HAADF-HRSTEM image simulation, such as the electron beam broadening due to interactions with low atomic number atoms from surfactants and the carbon support film, the residual sample tilt, residual optical aberrations, and any artifacts related to the scan stability or detection noise. However, the better agreement between the normalized peak intensity values from the experimental and simulated images in Figure 5b indicates that the correct assignment of fitting distributions has been achieved when estimating the thickness of each atomic column. The deviation noted for atomic column thicknesses below five atoms in Figure 5b is probably due to the stronger influence from the inherent noise of the detector and from the higher relative HAADF contribution from the lower atomic number elements in this ultra-thin regime. In addition, the electron beam focusing point relative to the sample topography and the defocus spread, which is not considered in the image simulation, may also contribute to this discrepancy.

It should be noted that the integrated peak intensity plot shown in Figure 5b includes error bars based on standard deviation measurements, which allow us to estimate the maximum error to be ±1 atom within a 90% confidence level.

## Conclusions

We describe a practical procedure to extract the approximate 3-D morphology of nanocrystals from single HAADF-HRSTEM images using the GMM classification approach. The specific surface areas from different facets of CeO_2_ nanoparticles were estimated after reconstruction of the 3-D morphology. The method is based on a mapping of column thicknesses with a high level of precision from single unfiltered images, in conjunction with the utilization of symmetry information pertinent to the unit cell of the material under investigation. The procedure is an alternative to tomographic reconstruction approaches [4,13,24,25] which tend to require a much greater experimental effort and may be prohibitive in practice due to electron beam sensitivity limitations of the nanocrystalline material under examination.

## Competing interests

The authors declare that they have no competing interests.

## Authors’ contributions

DGS, ERL, CJK, and AJR conceived the present study. MALC synthesized the samples. MALC, WW, and ERL performed the HRSTEM experiments. DGS, RDR, and LAM treated the HRSTEM data and applied the GMM analysis. DGS, LH, and JB carried out the image simulation procedures and analysis. All authors contributed significantly to the discussions and to the manuscript writing. All authors read and approved the final manuscript.

## Supplementary Material

Additional file 1 Supporting_information.pdf – Manuscript supporting information: peak detection and masking configurations; integrated intensity analysis from isolated Ce atoms; atomic structure files, videos, and interactive models from 3-D morphology models and symmetric model structures; HAADF-HRSTEM image calculation procedure and results. Click here for file

Additional file 2 **A_model_exp.cif – 3-D reconstruction structural files.** Atomic coordinates from reconstructed morphology of type-A nanocrystals. Click here for file

Additional file 3 **B_model_exp.cif – 3-D reconstruction structural files.** Atomic coordinates from reconstructed morphology of type-B nanocrystals. Click here for file

Additional file 4 **A_video_final.avi – Model visualization.** Video illustrating the reconstructed 3-D morphology of type-a nanocrystals and its comparison to the original images. Click here for file

Additional file 5 **B_video_final.avi – Model visualization.** Video illustrating the reconstructed 3-D morphology of type-B nanocrystals and its comparison to the original images. Click here for file

Additional file 6 **A_interactive_model.pdf – 3-D reconstruction models.** Interactive models of reconstructed morphologies from type-A nanocrystals. Click here for file

Additional file 7 **B_interactive_model.pdf – 3-D reconstruction models.** Interactive models of reconstructed morphologies from type-B nanocrystals. Click here for file

Additional file 8 **A_model_sym.cif – 3-D symmetric model for simulation.** Atomic coordinates from symmetric morphology of type-A nanocrystals used for the HRSTEM image simulation procedure. Click here for file

Additional file 9 **B_model_sym.cif – 3-D symmetric model for simulation.** Atomic coordinates from symmetric morphology of type-B nanocrystals used for the HRSTEM image simulation procedure. Click here for file
