# Supplementary material for: Assessment of a nanocrystal 3-D morphology by the analysis of single HAADF-HRSTEM images
Source: Nanoscale Res Lett. 2013 Nov 13;8(1):475. doi: 10.1186/1556-276X-8-475 (PMC3831815; doi:10.1186/1556-276X-8-475)
Supplement: Additional file 1 — Supporting_information.pdf – Manuscript supporting information: peak detection and masking configurations; integrated intensity analysis from isolated Ce atoms; atomic structure files, videos, and interactive models from 3-D morphology models and symmetric model structures; HAADF-HRSTEM image calculation procedure and results. [file 1556-276X-8-475-S1.pdf]

Supporting Information

Assessment of a nanocrystal 3D morphology by the analysis of single HAADF-HRSTEM images

Daniel G. Stroppa, Ricardo D. Righetto, Luciano A. Montoro, Lothar Houben, Juri Barthel, Marco A. L. Cordeiro, Edson R. Leite, Weihao Weng, Christopher J. Kiely, Antonio J. Ramirez

Content:

- Section S1 – Peak detection and masking algorithm
- Section S2 – Integrated intensity analysis from isolated Ce atoms
- Section S3 – Structural files, videos and interactive models from 3D morphology models and symmetric model structures
- Section S4 – HAADF-HRSTEM image calculation procedure and results

Section S1 – Peak detection and masking algorithm

Peak detection on the as-obtained HAADF-HRSTEM images was performed using a custom algorithm for detecting the atomic column positions by correlating the image with Gaussian masks of different radii. The integration mask radius was selected so as to capture approximately 80% of the peak signal. Such a configuration was chosen as it did not present overlaps between the signals from adjacent atomic columns and provided a reduced amount of inherent background noise during the peak integration.

Finally, a refinement of the center position for each atomic column was achieved by shifting the center of each mask within a ±2 pixel interval along the x and y directions. The center positions of atomic columns were considered to be the (x,y) coordinates with the maximum integrated value of pixels within the shifting range. Typical results from the peak detection procedure are presented in Figure S1.

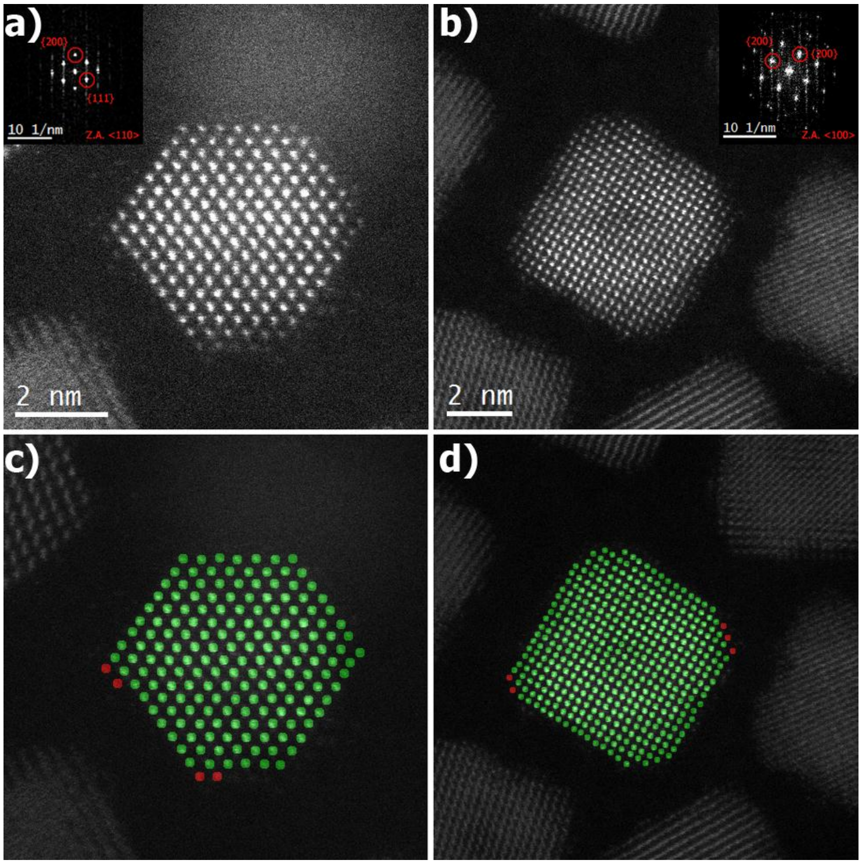

Figure S1. a, b) Experimental HAADF images from type-A and type-B nanocrystals, respectively. c, d) Superimposition of the integration masks over the corresponding experimental images after the refinement step. The masks indicated by the green color show which spots were used for the 3D morphology model. The masks indicated by the red color were used for verifying the assignment of the HAADF signal level from a single Ce atom during the GMM analysis.

Section S2 – Integrated intensity analysis from isolated Ce atoms

A preliminary inspection of HAADF-STEM images for type-A and type-B nanocrystals (Figures 1a and 1b) indicated that a few bright spots at the edge of the nanocrystal might possibly have arisen from a single or possibly two Ce atoms, as indicated by the red colored masks in Figures S1c and S1d. This supposition was based on the lack of symmetry of those positions in comparison with the well-developed surface facets of the nanocrystals.

The integrated values from these peripheral atoms were compared to the integrated intensity histograms in order to verify this single atom thickness assignment after the GMM fitting procedure. Figure S2 presents the intensity of the peripheral atoms and their spatial locations on the histograms according to the overall integrated intensity.

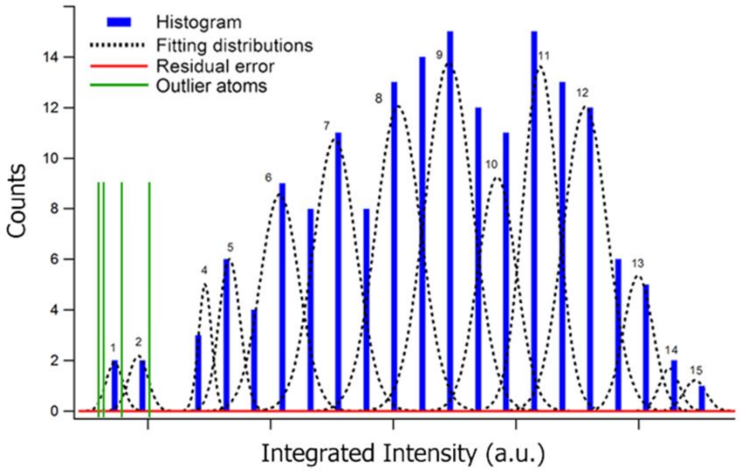

Figure S2. Intensity histogram and GMM fitting from nanocrystal A atomic columns. The integrated intensities from periphery atoms (see Figure S1c) are indicated as green lines.

As the integrated intensities* of the peripheral atoms are relatively close to the minimum atomic column intensity included in the 3D reconstruction, the assignment of the first fitting Gaussian curve to a single-atom atomic column is justified. Although a small discrepancy between the values from the GMM fitting for a single-atom atomic column and the peripheral atoms is acknowledged, its mean value is less than 55% of the average step between two adjacent fitting curves. Consequently, the integrated intensities from these peripheral atoms can be classified in the same fitting curve as the 'single atom atomic column' in the 3D morphology model.

* - except for the most intense one, which was assigned to the 2-atoms columns class.

Section S3 – Structural files, videos and interactive models from 3D morphology models and symmetric model structures

Both the 3D morphology models and symmetric model structures depicted in Figure S3 are available as plain text (.cif) files in the online supporting information. These files visualized/manipulated using various software packages, such as VESTA [1] and MEGACELL [2], respectively.

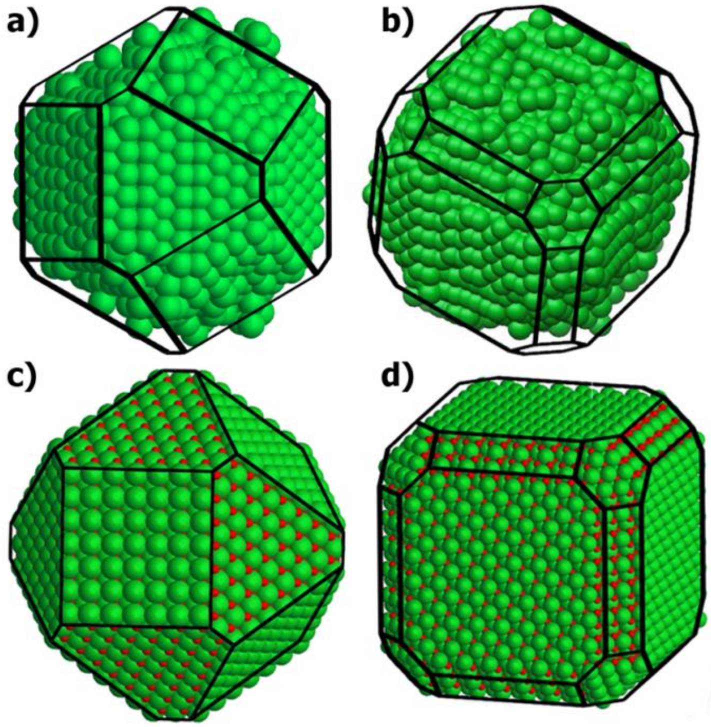

Figure S3. a,b) 3D morphology models and c,d) symmetric model structures from type-A and type-B nanocrystals after the HAADF-HRSTEM image analysis.

Interactive models and videos depicting the crystallographic structure from the 3D morphology models from both type-A and type-B nanocrystals can be found in the Additional files 4 to 9. The videos were generated with the aid of CHIMERA software [3].

1 – Available at http://www.geocities.jp/kmo_mma/crystal/en/vesta.html

2 – Available at http://megacell.lnls.br

3 – Available at http://www.cgl.ucsf.edu/chimera

Section S4 – HAADF-HRSTEM image calculation procedure and results

HRSTEM image simulations of the nanocrystal models were performed using the Dr. Probe software [4]. The multislice algorithm considered an incident 200kV electron wave emanating from a source with 0.07 nm radius, and passing through a 25 mrad radius condenser aperture. Spherical aberration (Cs) was the only optical aberration included in the calculation and this parameter was estimated to be 0.001 mm.

The structural files employed for both symmetric models A and B can be found in the on-line Supporting Information. The models A and B were sectioned into 29 and 41 equally distributed slices, respectively, aiming at single-atom layers for a better adequacy to the weak phase object approximation (WPOA). In order to include the effects of thermal motion, 10 frozen-phonon configurations were calculated for every slice. The multislice calculations for models A and B were performed for each pixel (scanning point) on the 298 x 298 and 256 x 256 image matrices, respectively. Hence similar pixel sizes were obtained in relation to the experimental images for nanocrystals A and B, respectively. The resultant wavefunction amplitudes were evaluated within a 110-300 mrad annular range.

Although the experimental HAADF-HRSTEM images presented well-faceted nanocrystals with a reasonably high degree of symmetry, a careful analysis after the counting of atomic rows indicate a few asymmetric features, as depicted in Figure S4.

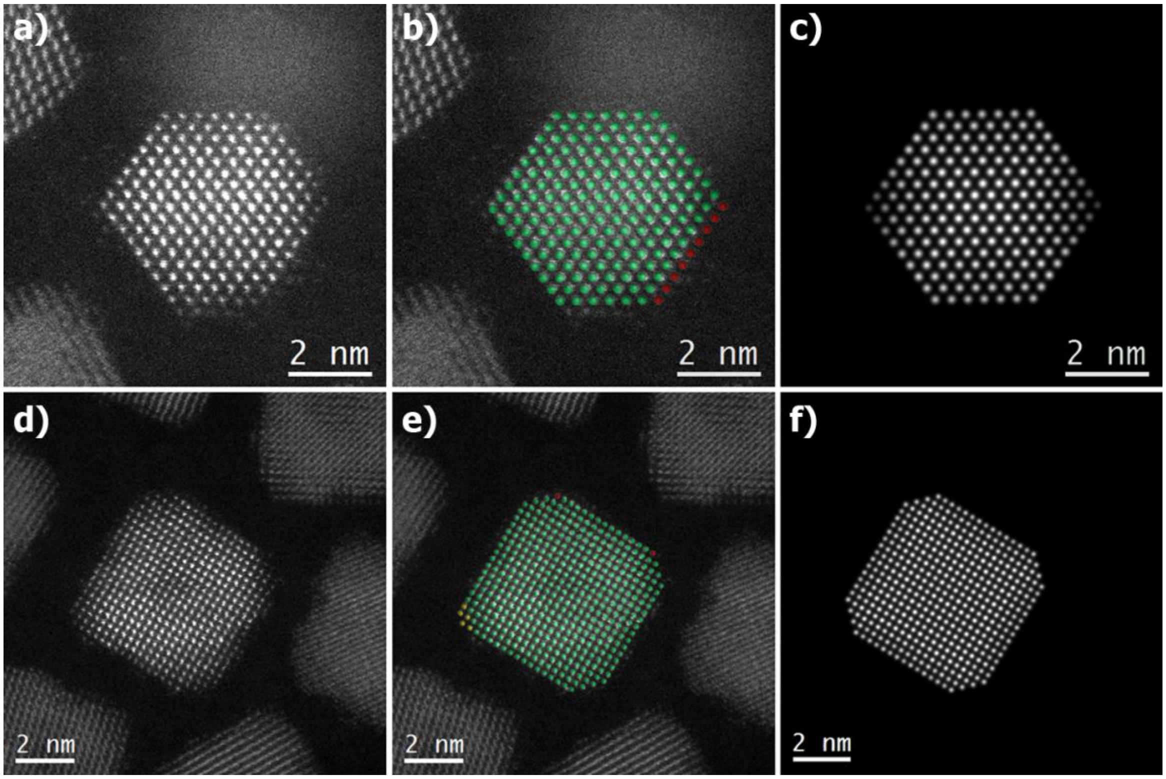

Figure S4. Qualitative comparison of a,d) experimental HAADF-HRSTEM images, b,e) symmetric models and c,f) simulated images from the symmetric models for nanocrystals A (row 1) and B (row 2), respectively. The atomic positions marked in red and yellow represent missing and extra atomic columns in the actual nanocrystals, respectively.
