# Supplementary figures and images for: Assessment of a nanocrystal 3-D morphology by the analysis of single HAADF-HRSTEM images
Source: Nanoscale Res Lett. 2013 Nov 13;8(1):475. doi: 10.1186/1556-276X-8-475 (PMC3831815; doi:10.1186/1556-276X-8-475)

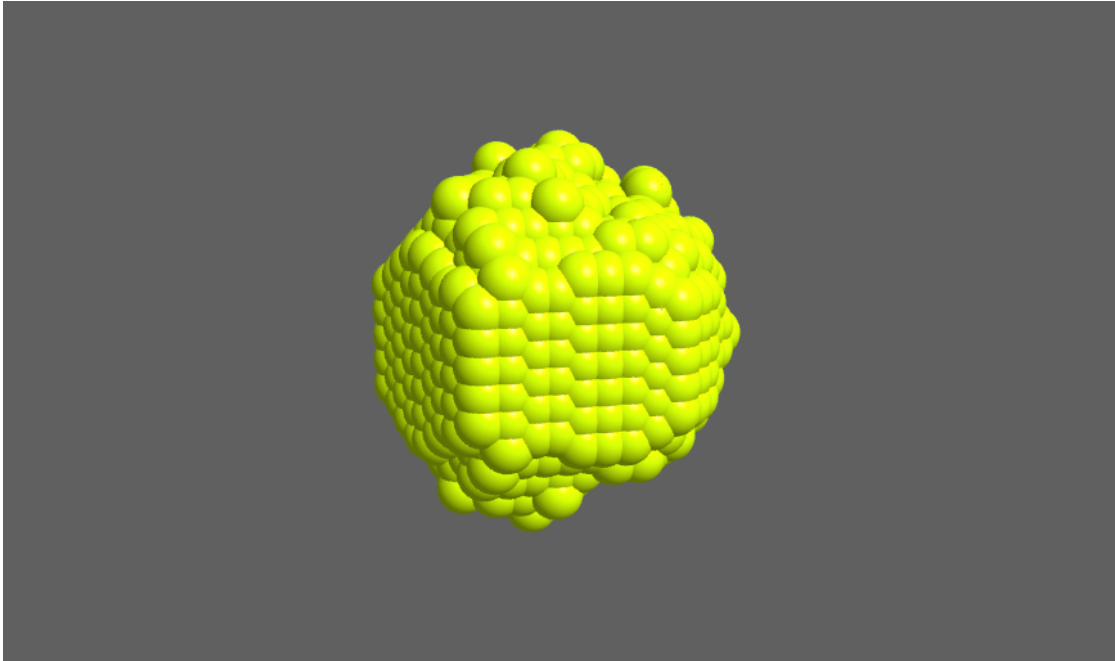

Supplement: Additional file 6 — A_interactive_model.pdf – 3-D reconstruction models. Interactive models of reconstructed morphologies from type-A nanocrystals. [file 1556-276X-8-475-S6.pdf]

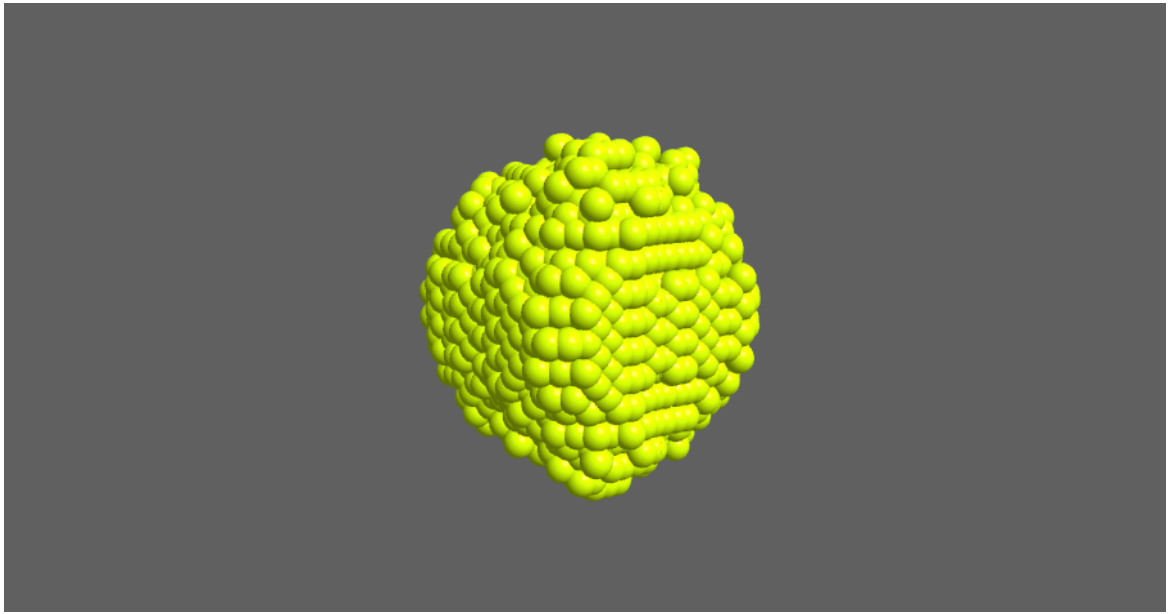

Supplement: Additional file 7 — B_interactive_model.pdf – 3-D reconstruction models. Interactive models of reconstructed morphologies from type-B nanocrystals. [file 1556-276X-8-475-S7.pdf]
